# Pentamidine Is Not a Permeant but a Nanomolar Inhibitor of the *Trypanosoma brucei* Aquaglyceroporin-2

**DOI:** 10.1371/journal.ppat.1005436

**Published:** 2016-02-01

**Authors:** Jie Song, Nicola Baker, Monja Rothert, Björn Henke, Laura Jeacock, David Horn, Eric Beitz

**Affiliations:** 1 Department of Pharmaceutical and Medicinal Chemistry, Christian-Albrechts-University of Kiel, Kiel, Germany; 2 Division of Biological Chemistry & Drug Discovery, School of Life Sciences, University of Dundee, Dundee, Scotland, United Kingdom; University of California, Los Angeles, UNITED STATES

## Abstract

The chemotherapeutic arsenal against human African trypanosomiasis, sleeping sickness, is limited and can cause severe, often fatal, side effects. One of the classic and most widely used drugs is pentamidine, an aromatic diamidine compound introduced in the 1940s. Recently, a genome-wide loss-of-function screen and a subsequently generated trypanosome knockout strain revealed a specific aquaglyceroporin, TbAQP2, to be required for high-affinity uptake of pentamidine. Yet, the underlying mechanism remained unclear. Here, we show that TbAQP2 is not a direct transporter for the di-basic, positively charged pentamidine. Even though one of the two common cation filters of aquaglyceroporins, i.e. the aromatic/arginine selectivity filter, is unconventional in TbAQP2, positively charged compounds are still excluded from passing the channel. We found, instead, that the unique selectivity filter layout renders pentamidine a nanomolar inhibitor of TbAQP2 glycerol permeability. Full, non-covalent inhibition of an aqua(glycero)porin in the nanomolar range has not been achieved before. The remarkable affinity derives from an electrostatic interaction with Asp265 and shielding from water as shown by structure-function evaluation and point mutation of Asp265. Exchange of the preceding Leu264 to arginine abolished pentamidine-binding and parasites expressing this mutant were pentamidine-resistant. Our results indicate that TbAQP2 is a high-affinity receptor for pentamidine. Taken together with localization of TbAQP2 in the flagellar pocket of bloodstream trypanosomes, we propose that pentamidine uptake is by endocytosis.

## Author Summary

Pentamidine is usable only in the early, non-cerebral stage of human African trypanosomiasis. An aquaglyceroporin, TbAQP2, is required for high-affinity pentamidine uptake. This study established the mechanism. Pentamidine does not pass TbAQP2 but blocks the unique selectivity filter via a single charged amidine and displacement of water from the channel, rendering it the first non-covalent, nanomolar aquaglyceroporin inhibitor. TbAQP2 does not act as a pentamidine transporter but a high-affinity receptor indicating drug uptake by endocytosis. The novel binding mode and proposed uptake mechanism open up possibilities for designing needed anti-trypanosomal small molecule drugs with regard to penetrating the blood-brain barrier for treatment of the severe, second disease stage.

## Introduction

A specific aquaglyceroporin, TbAQP2, is required for high-affinity uptake of pentamidine into human African trypanosomiasis parasites, *Trypanosoma brucei* [1–3]. Additionally, a trypanosome adenosine transporter mediates pentamidine transport, albeit at lower efficiency [2]. Aquaglyceroporins represent a subfamily of the aquaporin water channel proteins and conduct small, uncharged solutes, mainly glycerol and urea [4]. However, at a molecular weight of 340 Da and with two strongly basic, positively charged amidine moieties (pK_a_ 12.1), pentamidine is not a physiological substrate analog and differs from previous examples of drug uptake involving aquaglyceroporins [5,6]. In the treatment of acute promyelocytic leukemia, the human aquaglyceroporin AQP9 [7] serves as an entry site for the drug arsenic trioxide, As_2_O_3_ [8], which dissolves into weak arsenous acid, As(OH)_3_ (126 Da), resembling the glycerol molecule (92 Da). The analogous antimonous acid, Sb(OH)_3_ (173 Da), derived from the antimonial drug pentostam, is the first-line treatment of leishmaniasis and enters *Leishmania major* parasites via LmAQP1 [9]. The antineoplastic agent hydroxyurea (N-hydroxylated urea, 76 Da) is a permeant of PfAQP, i.e. the single aquaglyceroporin of the malaria parasite *Plasmodium falciparum*, and of TgAQP from the toxoplasmosis parasite *Toxoplasma gondii* [10].

**Fig 1:**
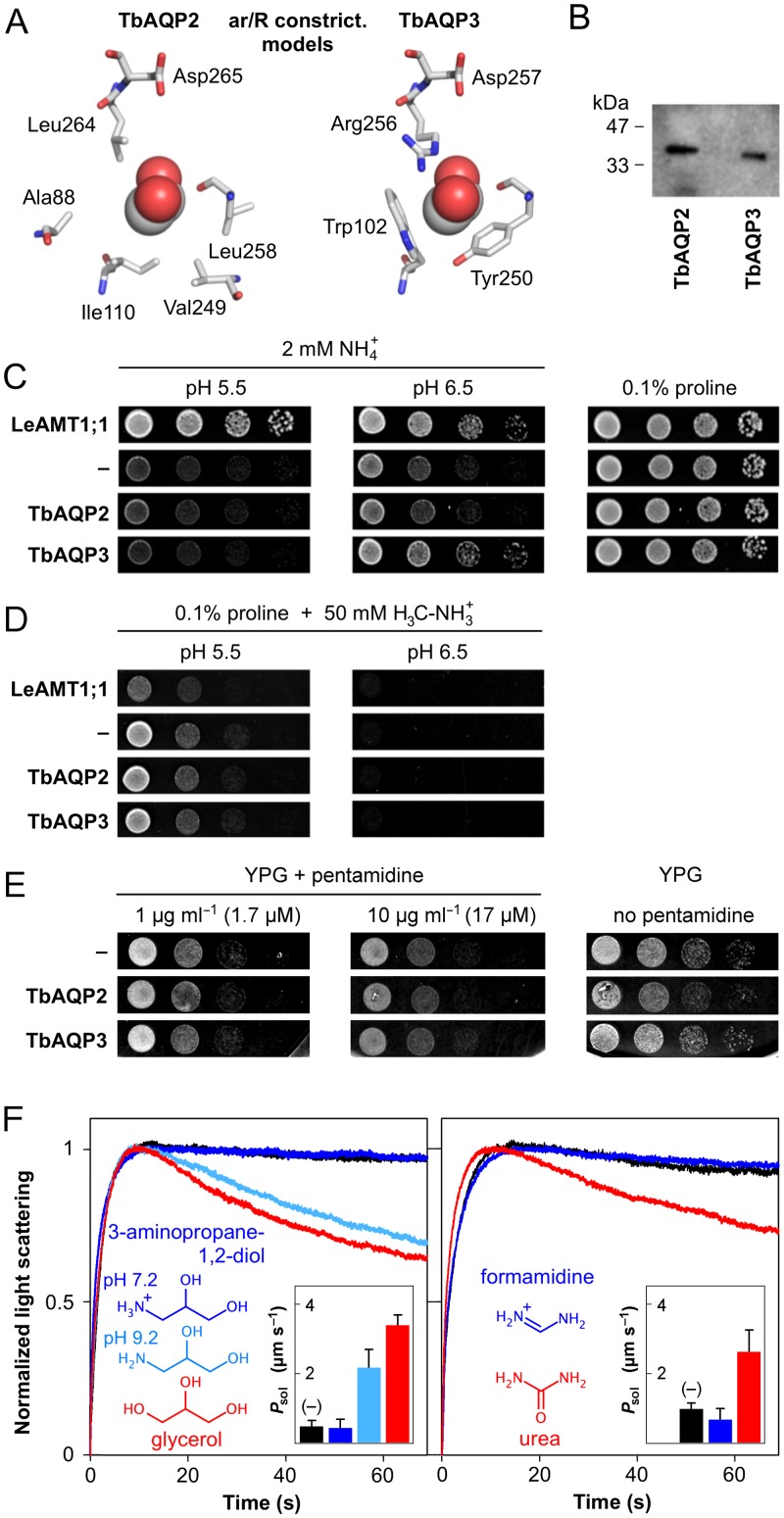
Cation exclusion by TbAQP2. (A) Shown is the layout of the aromatic/arginine selectivity filter of TbAQP2 (left) and TbAQP3 (right) as seen from the top. A bound glycerol molecule is shown as spheres. The models are based on the *E*. *coli* GlpF structure (PDB# 1FX8). (B) Western blot using membrane protein fractions isolated from yeast expressing TbAQP2 and TbAQP3 that were N-terminally tagged with a hemagglutinin epitope. (C) Phenotypic growth assay of 31019bΔmep1-3 yeast lacking endogenous ammonium transporters on media with NH_4_^+^ as the sole nitrogen source. Growth at pH 6.5 indicates mainly background transmembrane diffusion of uncharged NH_3_; growth at pH 5.5 is due to transport of charged NH_4_^+^. LeAMT1;1 is a tomato ammonium transporter, cells without transporter expression (–) served as negative controls. With proline as a nitrogen source growth is even for all cells. (D) Complementary phenotypic growth assay using yeast-toxic methylammonium instead of ammonium yielding opposite effects. (E) Expression of TbAQP2 does not increase the pentamidine-susceptibility of yeast grown on media with glycerol (YPG) as a non-fermentable carbon source. (F) Biophysical light scattering assays with TbAQP2-expressing yeast protoplasts in 300 mM hypertonic solute gradients. The rapid first phase of the traces indicates water efflux, followed by a slower solute influx phase. Red traces indicate permeability for physiological substrates glycerol (left) and urea (right). Traces of control cells without TbAQP2 are black. Structures and traces of 3-amino-propane-1,2-diol (left) and formamidine (right) are labeled dark blue (pH 7.2) or light blue (pH 9.2). The insets show deduced permeability coefficients (P_*sol*_) with S.E.M. from three independent experiments.

Trypanosomes express three aquaglyceroporins, TbAQP1-3, with permeability, among others, for glycerol, urea [11], arsenous and antimonous acid [12]. The physiological roles of TbAQP2-3 are unclear because the phenotype of knockout parasites is inconspicuous [2]. The physicochemical properties of pentamidine seem to be generally incompatible with a direct passage via AQPs, because two conserved filter sites select against positive charge and too large size. The selectivity filter or aromatic/arginine (ar/R) constriction is typically composed of an arginine residue in an aromatic environment (Fig 1A, right) and excludes solutes with diameters larger than 3–4 Å as well as protons [13–15]. The central Asn-Pro-Ala (NPA) filter is formed by the NPA-capped ends of two α-helices that emanate a positive electrostatic field and repel cations, such as sodium, potassium, and ammonium, NH_4_^+^ [14,16].

TbAQP2 contains a conserved ‘NPA/NPA’ cation filter with the crucial asparagines [16] intact and minor amino acid exchanges in the second and third position (NSA/NPS) [2,11]. In contrast, the ar/R constriction is virtually absent. Here, arginine is replaced by a neutral Leu264 and the common aromatic positions carry aliphatic residues (Ala88, Ile110, Val249, Leu258; Fig 1A) [2,11].

In this study, we asked whether pentamidine is a direct permeant of TbAQP2 and analyzed its interaction with the protein. We found that TbAQP2 is impermeable to even small cations, such as NH_4_^+^ or formamidine, and consistent with this, failed to increase the susceptibility of yeast cells to pentamidine. Strikingly, pentamidine bound avidly to the selectivity filter and fully inhibited TbAQP2 with an IC_50_ of 130 nM. This affinity is unprecedented in the on-going search for AQP inhibitors [17] and is derived from a unique interaction between one of the amidine moieties of pentamidine and Asp265 in conjunction with the displacement of water molecules from the channel interior. A single TbAQP2 point mutation, Leu264Arg, reversed pentamidine binding and rendered trypanosomes resistant. The localization of TbAQP2 in the flagellar pocket [2], characterized by high rates of membrane turnover, leads us to propose that pentamidine uptake occurs by endocytosis with TbAQP2 acting not as a transporter but a high-affinity pentamidine receptor. Such a mechanism could be exploited as a novel route to shuttle small molecule drugs into trypanosomes in a similar fashion as discussed for macromolecular transferrin protein-conjugated compounds in connection with the trypanosomal transferrin receptor [18,19].

## Results

### TbAQP2 excludes organic cations

To approach the question whether TbAQP2 is directly permeable for pentamidine, we set up a yeast expression system and initially analyzed permeability for small neutral and positively charged compounds. TbAQP3 carrying a conserved ar/R constriction (Fig 1A) served as a control. TbAQP2 and TbAQP3 were well expressed (Fig 1B) and functional as shown below.

First, we carried out phenotypic growth assays based on a yeast strain that lacks all three endogenous ammonium transporters (Δ*mep1-3*) [13,20]. This strain does not grow on acidic media with ammonium as the sole nitrogen source (Fig 1C, pH 5.5, second lane). The growth phenotype is pH-dependent due to the chemical equilibrium of NH_3_/NH_4_^+^ (pK_a_ of 9.2) and growth is permitted towards more alkaline pH by increasing transmembrane diffusion of neutral NH_3_. Permeability of transport proteins for charged NH_4_^+^ is indicated by growth at acidic pH, as seen with the tomato ammonium transporter 1;1 (LeAMT1;1) as a positive control (Fig 1C, top lane). In this assay, neither expression of TbAQP2 nor TbAQP3 enhanced the NH_4_^+^ uptake over that of cells without an ammonium transporter (Fig 1C). A complementary outcome of the transport assay can be obtained by using yeast-cytotoxic methylammonium, H_3_C-NH_3_^+^ [13]. Here, yeast growth will cease when the neutral methylamine form, H_3_C-NH_2_, diffuses across the cell membrane (Fig 1D, pH 6.5) or when the protonated, charged form is transported (Fig 1D, pH 5.5, top lane). This assay is particularly sensitive due to exposure of the cells to the toxic compound for several days [13]. Again, TbAQP2 and TbAQP3 appeared impermeable for the positively charged compound form and survived the treatment (Fig 1D). Pentamidine itself is cytotoxic to yeast when the cells are grown on a non-fermentable carbon source, such as glycerol [21]. Again, expression of TbAQP2 did not increase the susceptibility in comparison to non-expressing and TbAQP3 expressing cells (Fig 1E).

Next, we employed a biophysical light-scattering assay for TbAQP2 permeability [22]. We expressed TbAQP2 in a yeast strain that lacks the endogenous aquaglyceroporin (Δ*fps1*), prepared protoplasts, and challenged them by a hypertonic, test solute-containing buffer. In an initial rapid phase, the protoplasts will release water following the outward osmotic gradient and shrink as indicated by an increase in light scattering. In the second slower phase, volume will be regained if the test solute can pass TbAQP2 and enter the protoplasts. Pentamidine is not testable in this setup because its low solubility prohibits the establishment of a sufficiently concentrated gradient. We used instead two pairs of test solutes, glycerol/3-aminopropane-1,2-diol and urea/formamidine, of which glycerol and urea are established permeants of TbAQP2 [11], whereas the respective partners are bioisosteric nitrogen-derivatives, which are positively charged at neutral pH (Fig 1F). 3-Aminopropane-1,2-diol has a pK_a_ of 9.2; hence, at a buffer pH of 9.2, which yeast will tolerate for the assay time, 50% of the compound will be deprotonated and neutral. Formamidine is too strong a base (pK_a_ 13) to be amenable to deprotonation in aqueous solution. In the assay, TbAQP2 showed permeability for glycerol and urea (Fig 1F, red), whereas the positively charged compounds did not pass (Fig 1F, dark blue) as seen by traces equal to control protoplasts without TbAQP2 (Fig 1F, black). The lack of permeability for the charged compounds is not due to molecule size, because formamidine is even smaller than urea. Indeed, 3-aminopropane-1,2-diol, when partially neutralized by a pH shift to 9.2, passed TbAQP2 at two thirds the rate of glycerol (Fig 1F, light blue). We conclude that organic cations fail to traverse the TbAQP2 channel.

### Pentamidine is a nanomolar inhibitor of TbAQP2

Formally, formamidine represents one of the amidine moieties of pentamidine. We reasoned that if formamidine (44 Da) is excluded from passing TbAQP2, then the much larger pentamidine would be excluded as well. The lack of an increase in susceptibility of TbAQP2 expressing yeast to pentamidine further argues against pentamidine transport via TbAQP2. Consequently, we conducted another set of light scattering assays to determine how pentamidine interacts with TbAQP2.

**Fig 2:**
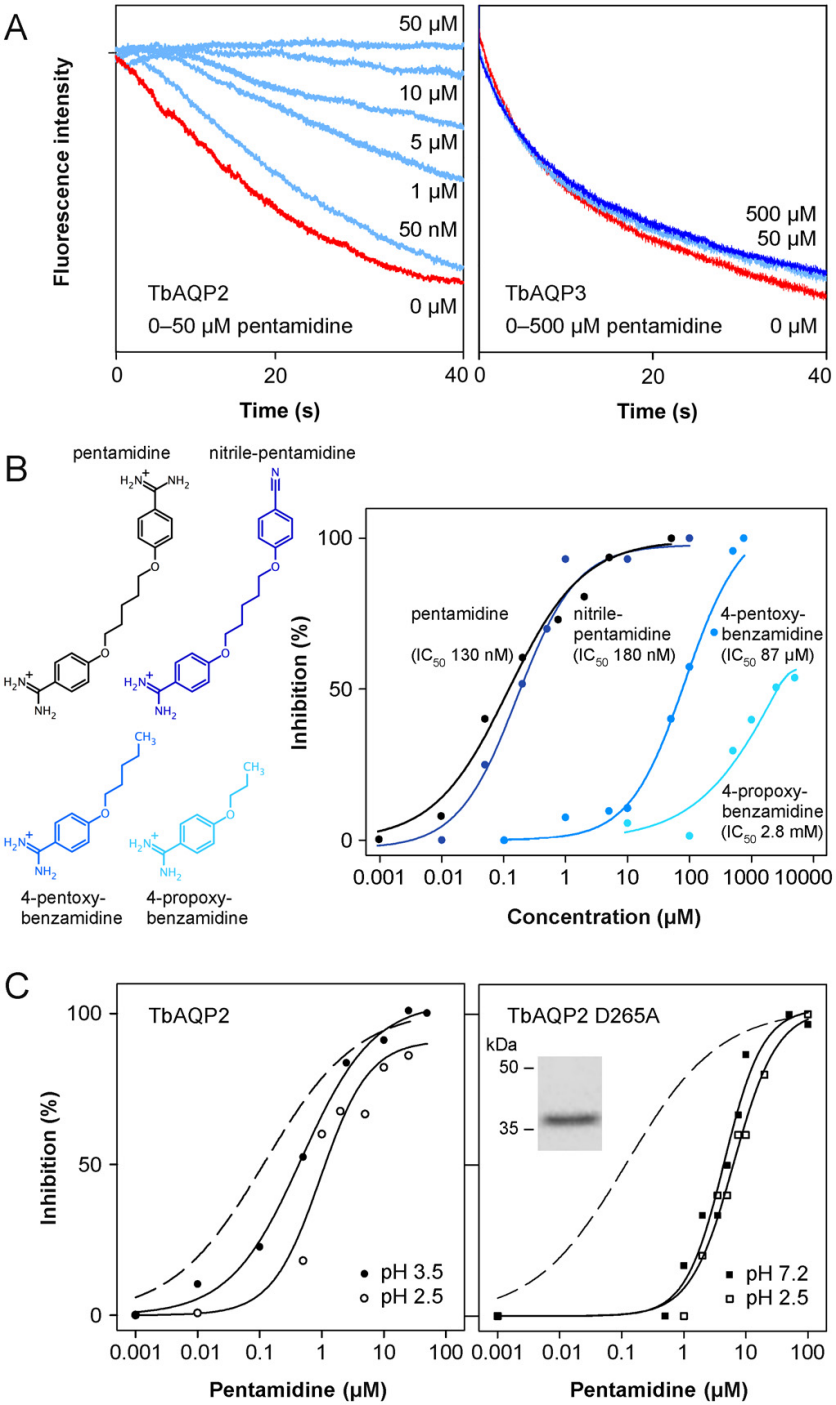
Inhibition of TbAQP2 glycerol permeability by pentamidine and derivatives. (A) Shown are example traces of protoplast light scattering in 300 mM isotonic glycerol gradients in the presence of pentamidine. Red traces indicate uninhibited glycerol influx via TbAQP2 (left) and TbAQP3 (right), effects of pentamidine concentrations from 50 nM to 50 μM are colored light blue, and of 500 μM pentamidine in dark blue. (B) Structure-function evaluation of TbAQP2 inhibition by pentamidine. The chemical structures of the used compounds are shown on the left and respective dose-response curves on the right. (C) Effect of pH titration of TbAQP2 Asp265 and replacement by mutation to alanine on pentamidine inhibition. The dashed lines show the pentamidine inhibition of wild-type TbAQP2 at pH 7.2 (data taken from Fig 2B). The left panel shows dose-response curves of pentamidine pH 3.5 (closed symbols; IC_50_ 450 nM), and pH 2.5 (open symbols; IC_50_ 1.1 μM). The effect of the Asp265Ala point mutation (expression confirming Western blot in the inset) on the inhibition by pentamidine is depicted on the right. Data taken at pH 7.2 (IC_50_ 4.5 μM) are indicated by closed symbols, those at pH 2.5 (IC_50_ 5.8 μM) by open symbols. Each data point is an average of 5–9 light scattering traces each from at least two independent experiments.

We switched from hypertonic assay conditions to an isotonic glycerol replacement of 300 mM saccharose from the buffer to omit osmotic water permeability effects and to obtain monophasic solute-uptake traces, which allow for a more accurate quantification especially at low rates [23]. The red traces in Fig 2A show unhindered glycerol influx via TbAQP2 and TbAQP3. Addition of up to 500 μM pentamidine 10 min prior to the assay left the glycerol permeability of TbAQP3 unaffected, whereas TbAQP2 was fully blocked already at 50 μM (Fig 2A). The inhibition of TbAQP2 by pentamidine was dose-dependent and substantial even in the nanomolar concentration range. We translated the relative glycerol permeability rates into a dose-response curve yielding an IC_50_ of 130 nM (Fig 2B). Pentamidine was effective immediately after addition to the protoplasts indicating that the binding site is readily accessible from the extracellular space.

### Pentamidine binds to Asp265 of TbAQP2 and displaces water from the channel

Since high-affinity inhibition of other AQPs is only achieved by covalent cysteine-modification by metalorganic compounds, e.g. mercurials or gold-containing auphen (IC_50_ 0.8 μM) [17,24], we set out to investigate the binding mode of pentamidine by determining structure-function relationships and by point mutation of TbAQP2.

We synthesized asymmetric variants of pentamidine by replacing one of the positively charged amidine moieties by nitrile, and by shortening the molecule via 4-pentoxy-, 4-propoxy- to 4-methoxy-benzamidine (Fig 2B). The nitrile compound was as effective as pentamidine in blocking TbAQP2 glycerol permeability (IC_50_ 180 nM) showing that one positive site is sufficient for full inhibition, most likely by electrostatical interaction with an acidic residue of TbAQP2. Removal of one benzamidine group (4-pentoxy-benzamidine) led to a shift in IC_50_ to 87 μM (Fig 2B). Further shortening of the molecule to 4-propoxy-benzamidine resulted in an IC_50_ of 2.8 mM and a maximal inhibition of 60%. The methoxy compound was incapable of inhibiting TbAQP2. Since electrostatic interactions exhibit highest affinity when efficiently shielded from water, our structure-function evaluation suggests that the amidine interaction site is located 7–8 Å, i.e. the length of the pentoxy chain, away from the solvent into the channel. This distance coincides with the position of the ar/R selectivity filter region.

TbAQP2 lacks the conserved arginine in the ar/R filter possibly providing access of the acidic Asp265 to the channel surface (Fig 1A). If the Asp265 sidechain carboxylate (pK_a_ 3.7 in water) interacts with pentamidine, the affinity should be titratable by pH. Hence, we determined inhibition of TbAQP2 by pentamidine at pH 3.5 and 2.5 and observed 3 times (450 nM) and 8 times (1.1 μM) reduced affinity, respectively (Fig 2C, left). To test whether the pH titration is specific to Asp265, we changed the residue by point mutation to alanine, which equally led to a shift in IC_50_ to 4.5 μM (Fig 2C, right). Further, this mutant was pH-independent with regard to pentamidine binding (IC_50_ 5.8 μM at pH 2.5; Fig 2C, right) confirming the identity of the interaction site.

Together, the structure-function evaluation and the mutation data indicate that the exceptional affinity of pentamidine to TbAQP2 is derived from an electrostatic interaction of one amidine moiety with Asp265 and shielding from the solvent by displacement of water molecules from the channel by the pentoxy chain.

### Mutation of Leu264 to arginine prevents pentamidine from binding to TbAQP2 and renders parasites resistant

A selectivity filter arginine followed by an aspartate at the next sequence position is a signature motif of aquaglyceroporins [25]. At about 4.5 Å distance in the protein, both charged residues are positioned for an electrostatic interaction, see for instance TbAQP3 Arg256/Asp257 in Fig 1A [26]. Besides TbAQP2, there are other examples of ar/R selectivity filters in which the arginine is replaced by an aliphatic residue, e.g. the *T*. *gondii* TgAQP or the AQP subfamily of plant tonoplast instrinsic proteins TIP2;1 [10]. However, in all such cases, replacement of the arginine goes together with an exchange of the neighboring aspartate for a neutral residue [27]. TbAQP2 is, thus, unique in having lost the arginine but having kept Asp265. We concluded that a point mutation of TbAQP2 Leu264 to arginine should re-establish the internal salt bridge with Asp265 and prevent pentamidine from binding.

**Fig 3:**
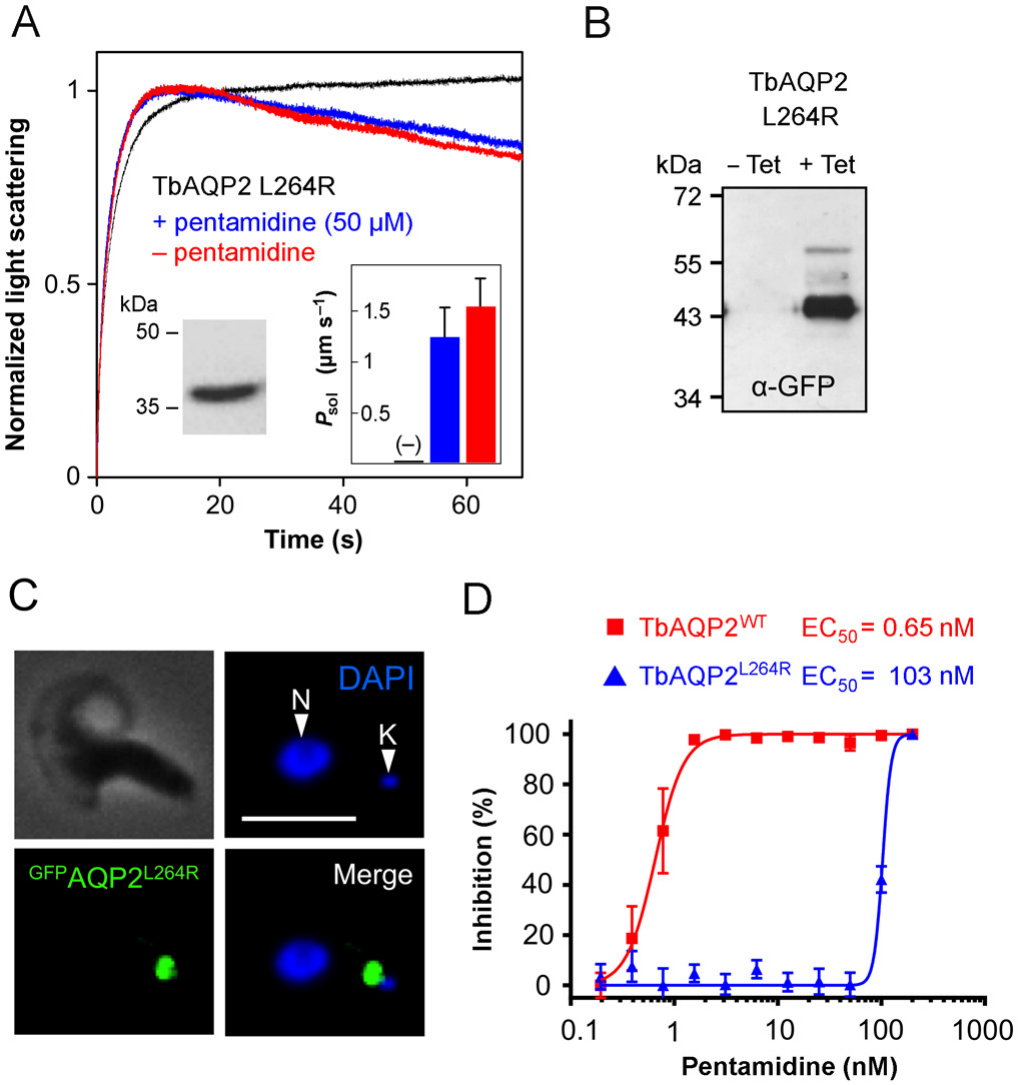
Loss of pentamidine inhibition and gain of resistant parasites by a TbAQP Leu264Arg mutation. (A) Light scattering assay with yeast protoplasts expressing TbAQP2 Leu264Arg in a 300 mM hypertonic glycerol gradient. Data taken in the absence of pentamidine are labeled red, those taken in the presence of 50 μM pentamidine are blue, and protoplasts without an AQP are black. The error bars denote S.E.M. from three independent experiments. (B) Western blot analysis showing inducible expression of GFP-TbAQP2 Leu264Arg (^GFP^AQP2^L264R^). (C) Immunofluorescence microscopy analysis of *T*. *brucei* expressing ^GFP^AQP2^L264R^. Scale-bar, 5 μm. (*D*) Dose–response curve for pentamidine in *aqp2*-null stains re-expressing wild-type GFP-TbAQP2 or ^GFP^AQP2^L264R^. EC_50_ values are indicated. Error bars indicate SD from two independent clones assessed in triplicate assays.

Expression in yeast of TbAQP2 Leu264Arg yielded a functional water and glycerol channel with about half the permeability of the wild-type protein (Fig 3A). Importantly, pentamidine failed to inhibit TbAQP2 Leu264Arg (Fig 3A) and this further supported the binding mode suggested above. We next investigated the findings from the heterologous yeast expression system in *T*. *brucei* parasites by expressing GFP-tagged TbAQP2 Leu264Arg (^GFP^AQP2^L264R^) or wild-type TbAQP2 (^GFP^AQP2^WT^) in a *T*. *brucei aqp2*-null background (Fig 3B). Despite the mutation, TbAQP2 Leu264Arg localized to the flagellar pocket as reported previously for wild-type TbAQP2 [2] (Fig 3C). While *aqp2*-null *T*. *brucei* expressing wild-type TbAQP2 were highly sensitive to pentamidine (EC_50_ = 0.65 nM), *T*. *brucei* expressing mutant TbAQP2 remained resistant to pentamidine (EC_50_ = 103 nM; Fig 3D). Thus, TbAQP2 Leu264Arg fails to bind pentamidine and fails to sensitize trypanosomes to pentamidine.

## Discussion

Maintenance of cation homeostasis is crucial for cell functionality and viability [14]. Accordingly, cation channels and transporters are highly regulated and/or selective, whereas non-cation conducting membrane proteins contain exclusion filters. The typical AQP channel layout exhibits two cation filters, which select against both types of cation conductance: a) hydrogen-bonded proton-wires are disrupted in the ar/R constriction preventing the Grotthuss mechanism, b) diffusion of non-proton cations is blocked by the electrostatic field generated in the NPA region [13–16]. Further, the amphipathic channel lining of the AQPs provide coordinating carbonyl oxygens only on one side, and therefore cannot sufficiently compensate for the dehydration penalty derived from the removal of the water shell from the ion [28]. Finally, the AQP protein structure is rigid and does not allow for conformational changes, because even small bends would obstruct the long (20 Å) and narrow (3–4 Å) water and solute channel [26], forbidding transporter-type cation transduction following the alternate access mechanism [29]. As a consequence, naturally occurring AQPs with cation conductance along the water/glycerol channel have not been identified [28]; there is evidence, though, for cation permeability via the central pore of the human AQP1 tetramer [30].

**Fig 4:**
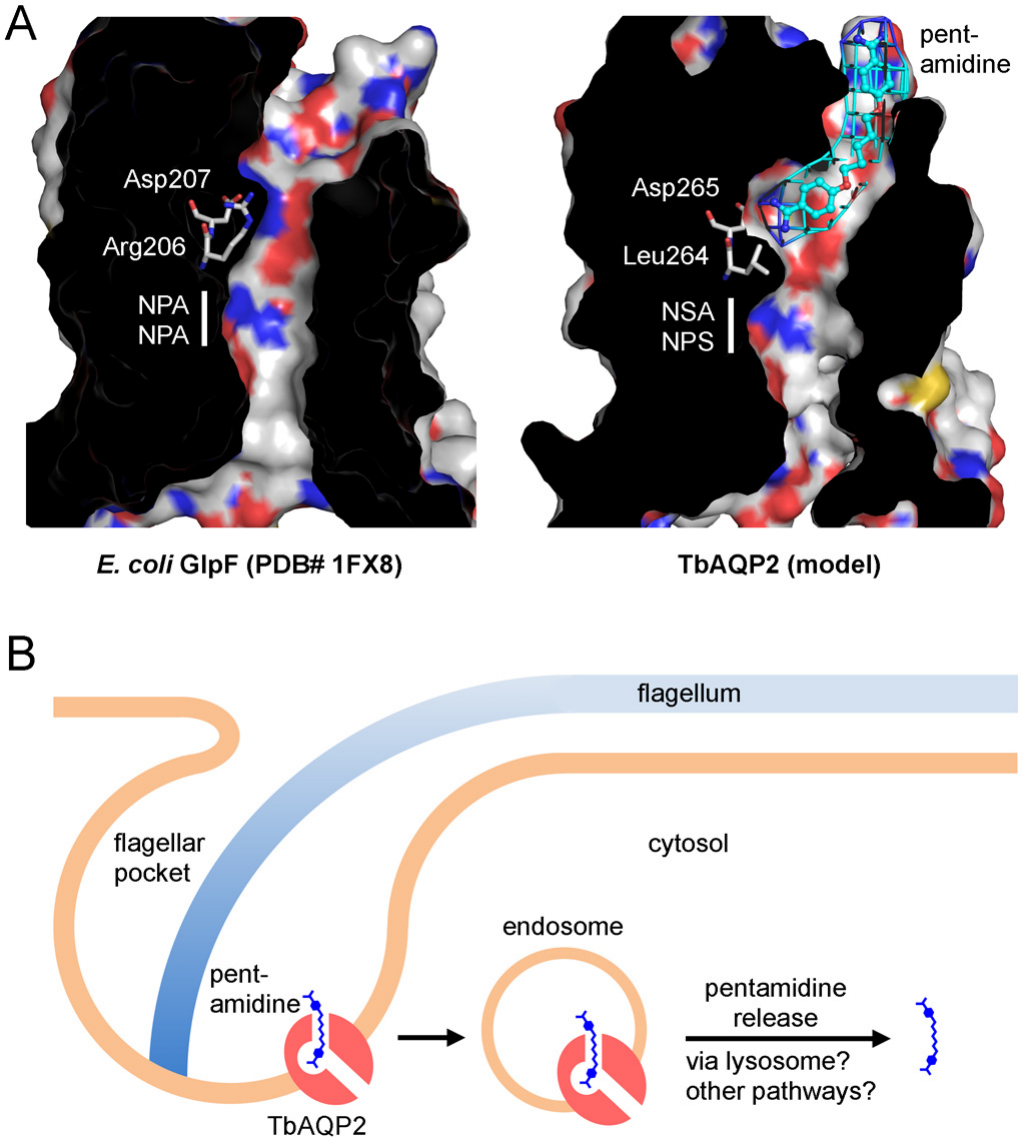
Model of the pentamidine binding mode to TbAQP2 and proposed uptake by endocytosis in the flagellar pocket. (A) Shown are the crystal structure of the prototypical aquaglyceroporin GlpF and a model of TbAQP2. GlpF Arg206 and TbAQP2 Leu264 mark the position of the ar/R selectivity filter. In TbAQP2, the Asp265 sidechain carboxylate binds to an amidine moiety of pentamidine (light blue), whereas in GlpF the space is occupied by the guanidine sidechain of Arg206. The location of the ‘NPA/NPA’ region (white bar) and sequence deviations in TbAQP2 are indicated. (B) Proposed uptake mechanism of pentamidine via high-affinity binding to TbAQP2, endocytosis of the complex, and release of pentamidine in the acidic lysosome due to pH shift or TbAQP2 degradation.

TbAQP2 lacks an ar/R filter, which could potentially allow for proton leakage. The NPA region, however, is intact and perfectly repels cations, such as NH_4_^+^ and charged formamidine, as shown in this study. Thus, pentamidine containing two formamidine substructures cannot pass TbAQP2. Instead, pentamidine acts as a high-affinity binder and inhibitor of TbAQP2 with one amidine moiety interacting with Asp265 by taking the place of the lacking arginine sidechain. The electrostatic interaction is increased by several orders of magnitude due to the exclusion of water from the channel (see Fig 4A for a proposed binding mode). Inhibition of TbAQP2 water and solute permeability is not the mode of pentamidine action because parasites lacking the aquaglyceroporin thrive well [2].

The obtained high affinity of 130 nM despite a steep glycerol gradient of 300 mM further argues against pentamidine diffusion through TbAQP2, because even high-affinity, alternate access-type transporters typically exhibit weaker, micromolar binding to enable release of the cargo after traversing the membrane [29]. AQP channel-like transporters, such as the malaria parasite’s lactate transporter, PfFNT, or the bacterial formate transporter, FocA, show K_m_ values in the high millimolar range, i.e. 87 mM and 96 mM, respectively [31,32]. In fact, nanomolar binding constants are typically associated with high-affinity receptors.

If TbAQP2 acts as a receptor rather than a transporter for pentamidine, how could drug uptake be achieved? TbAQP2 is specifically localized to the flagellar pocket of bloodstream *T*. *brucei* parasites, which is characterized by high membrane turnover activity [33]. This activity is indispensable for the parasite and required, among others, for immune evasion and nutrient uptake. From our data we conclude that a likely scenario for TbAQP2-dependent, high-affinity uptake of pentamidine into trypanosomes is via continued, non-selective endocytosis of pentamidine tethered to TbAQP2 as a receptor (Fig 4B). The hypothesis of pentamidine binding to TbAQP2 and subsequent uptake by endocytosis is strikingly supported by observations from the original biochemical characterization of pentamidine uptake [34]. Key findings were: a) a single amidine moiety suffices to initiate high-affinity transport, which is in agreement with the binding mode of pentamidine to TbAQP2 (Fig 4A), b) transport is mainly unidirectional and highly concentrative, and c) the process requires metabolic energy; the latter two points are fully compatible with endocytosis. Moreover, it is intriguing that in the procyclic, insect form of the parasites TbAQP2 is distributed over the entire surface of the plasma membrane [2] and these cells were found to transport slower and accumulate less pentamidine [2,35] further pointing at the importance of processes in the flagellar pocket for efficient pentamidine uptake. Intracellular release of pentamidine from TbAQP2 may be triggered downstream by acidification of the endo-lysosomal system that weakens the interaction with Asp265 (Fig 4B).

It is becoming increasingly clear that highly active processes in the flagellar pocket of African trypanosomes can be exploited for therapeutic purposes [33,36,37]. The classic pentamidine therapy appears to have been doing just that for decades.

Future routes in drug design could foresee fusion scaffolds that contain a minimal pentamidine anchor, e.g. alkoxy-benzamidine, for TbAQP2 binding linked to an anti-trypanosomal cargo to be delivered into the parasite. There is a good chance for achieving sufficient specificity because of the unique TbAQP2 ar/R filter composition. This approach would be similar to the idea of generating drug fusions with the transferrin protein and transport via endocytosis of the transferrin receptor in the flagellar pocket [19]. A recent study targeted pentamidine-loaded chitosan nanoparticles via a single domain nanobody to the parasite surface, inducing endocytotic uptake [38]. Addressing TbAQP2, however, would have the advantage that small molecules could be drafted, which, contrary to pentamidine, require only one positive moiety for Asp265 binding. Using small molecules increases the chance that the compounds will cross the blood brain barrier, which is a necessity to treat the severe cerebral stage of trypanosomiasis.

## Materials and Methods

### Plasmid constructs and western blots

TbAQP2, TbAQP2-L264R, TbAQP-D265A, and TbAQP3 were cloned via BamH I and HinD III into the pRS426Met25 vector [39]. The constructs carry an N-terminal hemagglutinin (HA) epitope-tag for detection by Western blot using a monoclonal mouse anti-HA antibody (1: 5000, Roche) and a horseradish peroxidase secondary anti-mouse antibody (1: 2000, Jackson Immuno Research) for ECL detection (Amersham). For *T*. *brucei* expression, the pRPa^GFPx^ construct [1] was modified to express GFP-tagged AQP2 under the control of a Tet-regulated *RRNA* promoter. Site directed mutagenesis was carried out using the QuikChange Multi Site-Directed Mutagenesis Kit (Agilent technologies). Point mutations were confirmed by sequencing. Primer sequences are available upon request. *T*. *brucei* whole cell lysates were stored in the presence of a protease inhibitor cocktail (Roche) and were not boiled. GFP was detected using polyclonal rabbit α-GFP (Europa; 1:2000).

### Phenotypic yeast growth assays

The *Saccharomyces cerevisiae* strain 31019bΔmep1-3 (MATa ura3 mep1Δ mep2Δ::LEU2 mep3Δ::KanMX) was used, which lacks all endogenous ammonium transporters [40]. Cells expressing TbAQP2, TbAQP3, tomato AMT1;1 [41], or no channel were grown overnight in liquid minimum YNB-glucose medium (0.17% YNB supplemented with 2% glucose, 0.5% (NH_4_)_2_SO_4_, 2 × 10^−3^% histidine, 2 × 10^−3^% lysine, 0.01% leucine, Difco). Cultures were adjusted to an OD600 of 1, and diluted in a series of 1:10 steps. For NH_4_^+^ uptake, 5 μl cell suspensions were spotted on agar plates of 20 mM MES-buffered (pH 5.5 and pH 6.5) YNB-glucose medium containing 2 mM (NH_4_)_2_SO_4_ as the sole nitrogen source. Plates containing 0.1% proline were used for monitoring normal growth. For methylammonium uptake, cells were grown in YNB-glucose medium supplemented with 0.1% proline as a nitrogen source and 50 mM methylammonium (Sigma). Susceptibility for pentamidine was assayed by growth on YPG agar media (1% yeast extract, 2% peptone, 2% glycerol) without or with addition of pentamidine isethionate at 1 μg ml^–1^ (1.7 μM) or 10 μg ml^–1^ (17 μM) [21]. Growth was monitored for 2–5 days.

### Biophysical protoplast light scattering assays

The *S*. *cerevisiae* strain BY4742Δfps1 *(MATa*, *his3-1*, *leu2Δ0*, *lys2Δ0*, *ura3Δ0*, *yll043w*::*KanMX*) lacking the endogenous aquaglyceroporin Fps1 was obtained from Euroscarf, Frankfurt. Yeast cells expressing TbAQP2, TbAQP2-L264R, TbAQP-D265A, TbAQP3, or no channel were collected at 4000 × g and 4°C, washed, and incubated for 15 min in 3 ml phosphate buffer (50 mM, pH 7.2) plus 0.2% 2-mercaptoethanol. 6 ml of phosphate/2-mercaptoethanol buffer containing 1.8 M saccharose, 200 units of Zymolyase-20T (MP Biomedicals, Illkirch, France), and 100 mg BSA Fraction V (Roth, Karlsruhe, Germany) were added and the suspension was incubated on an orbital shaker at 100 rpm for 60 min at 29°C. Protoplasts were collected at 2000 × g and 4°C, washed, and resuspended in 3 ml buffer (10 mM MOPS for pH 7.2; citric acid for pH 2.5 and 3.5, or Tris for pH 9.2) plus 1.2 M saccharose, 50 mM NaCl, and 5 mM CaCl_2_. For the assay, protoplast suspensions were diluted to an OD_600_ of 2. All measurements were done using a stopped flow apparatus (SFM-300, BioLogic, Claix, France) with a dead time around 10 ms, total flow rate of 14 ml/s, total volume of 202 μl, at 20°C. For hypertonic solute permeability assays, protoplasts were rapidly mixed with the same volume of buffer supplemented with 600 mM solute (glycerol, urea, 3-amino-1,2-diol, or formamidine), generating a 300 mM hypertonic solute gradient. For isotonic glycerol permeability measurements, protoplasts were mixed with the same volume of buffer in which 0.6 M saccharose were replaced by glycerol, generating an isotonic 300 mM glycerol gradient. Pentamidine and derivatives were added 10 min prior to the assay. Protoplast volume changes were monitored by measuring the intensity of 90° light scattering at 546 nm. In each experiment, 6 to 9 single curves were averaged, exponentially (rapid rates) or linearly fitted (slow rates) and the resulting time constants were related to solute permeability. The solute permeability coefficient *P*_*sol*_ was calculated using *P*_*sol*_ = |*dI*/*dt*| · (*V*_*0*_ · *C*_*out*_)/(*S*_*0*_ · *C*_*diff*_) [16], with *dI/dt* being the slope of the intensity curve, *V*_0_ the initial mean protoplast volume (65.45 μm^3^), *S*_*0*_ the initial mean protoplast surface area (78.54 μm^2^), *C*_*out*_ is the total external solute concentration (1.5 M), and *C*_*diff*_ is the chemical solute gradient (0.3 M).

### Pentamidine derivatives

Pentamidine and 4-methoxy-benzamidine were from Sigma. 4-Pentoxy- and 4-propoxy-benzamidine were generated by Williamson ether synthesis from 4-hydroxy-benzonitrile and 1-bromopentane or 1-bromopropane, respectively, and subsequent conversion of the nitrile to amidine using CH_3_Al(Cl)NH_2_ [42]. The asymmetric pentamidine derivative carrying one amidine and one nitrile moiety was generated in a first step by Williamson ether synthesis of 4-hydroxy-benzonitrile and 1,5-dibromopentane to obtain the di-nitrile compound. The second step was done according to Pinner [43] in molecular sieve-dried ethanol using HCl gas as an acidic catalyst and stopped before full conversion of the nitriles to imino esters. The semi-converted product was isolated by chromatography and the single amidine group was obtained by reaction with gaseous ammonia in ethanol. Nitrile-pentamidine hydrochloride: ^1^H NMR (300 MHz, DMSO-d_6_) δ 9.33 (2H, s), 9.19 (2H, s), 7.88 (2H, d), 7.74 (2H, d), 7.11 (4H, m), 4.09 (4H, m), 1.8 (4H, m), 1.57 (2H, m).

### Trypanosome cultures

Bloodstream-form *T*. *brucei*, Lister 427, MiTat 1.2, clone 221a and derivatives were maintained as previously described [2]; 2T1 [1] and *aqp2* null strains [2] were also described previously. Strains were transfected using a Nucleofector apparatus (Lonza) in conjunction with cytomix and transformants were selected with hygromycin (2.5 μg ml^–1^). ^GFP^AQP expression was induced by exposing cells to 1 μg ml^–1^ tetracycline for 48 h. EC_50_ assays were carried out using alamarBlue as described [44,45].

### Microscopy

*T*. *brucei* immunofluorescence microscopy was carried out according to standard protocols. Briefly, cells were settled on slides and mounted in Vectashield (Vector Laboratories) containing the DNA counterstain, 4,6-diamidino-2-phenylindole (DAPI). Images were captured using a Nikon Eclipse E600 epifluorescence microscope in conjunction with a Coolsnap FX (Photometrics) charge-coupled device (CCD) camera and processed in Metamorph 5.0 (Photometrics).

### Accession numbers

*Trypanosoma brucei* AQP2: CAG27021 (GenBank)

*Trypanosoma brucei* AQP3: CAG27022 (GenBank)

*Lycopersicon esculentum* AMT1;1: P58905.1 (SwissProt)

*Saccharomyces cerevisiae* MEP1: CAA97132 (GenBank)

*Saccharomyces cerevisiae* MEP2: CAA96025 (GenBank)

*Saccharomyces cerevisiae* MEP3: DAA11552 (GenBank)

*Saccharomyces cerevisiae* Fps1: CAA38096 (GenBank)

*Escherichia coli* GlpF: BAE77383 (GenBank)
